# Efficacy of Artemether-Lumefantrine on various *Plasmodium falciparum Kelch 13* and *Pfmdr1* genes isolated in Ghana

**DOI:** 10.1016/j.parepi.2020.e00190

**Published:** 2020-10-26

**Authors:** Enoch Aninagyei, Comfort Dede Tetteh, Martin Oppong, Alex Boye, Desmond Omane Acheampong

**Affiliations:** a University of Health and Allied Sciences, School of Basic and Biomedical Sciences, Department of Biomedical Sciences, PMB 31, Ho-Volta Region, Ghana; b Ghana Health Service, Municipal Health Directorate, Ga West Municipal, Amasaman, Ghana; c University of Cape Coast, School of Allied Health Sciences, Department of Medical Laboratory Science, Cape Coast, Ghana; d University of Cape Coast, School of Allied Health Sciences, Department of Biomedical Sciences, Cape Coast, Ghana

**Keywords:** Artemether-Lumefantrine, Parasite clearance characteristics, *Kelch 13* gene mutations, *Pfmdr1* genes, Ga West Municipal, Ghana, A, alanine, ACT, Artemisinin-based Combination Therapy, A-L, Artemether-Lumefantrine, AS-AQ, Artesunate-Amodiaquine, CRC, clearance rate constant, DHAP, Dihydroartemisinin-Piperaquine, dsDNA, double stranded DNA, G-6-PD, Glucose-6-phosphate dehydrogenase, GHS, Ghana Health Service, PCTs, parasite clearance times, *Pfmdr1*, Plasmodium multidrug resistance gene, SNPs, Single nucleotide polymorphisms, sWGA, selective whole genome amplification, WHO, World Health Organization, Amino acids:, A-alanine, C, cysteine, D, aspartic acid, *F*, phenylalanine, G, glycine, I, isoleucine, N, asparagine, V, valine, Y, tyrosine

## Abstract

**Introduction:**

Artemether-Lumefantrine (A-L) remains the drug of choice for the treatment of uncomplicated malaria in Ghana. However, the pharmaco-activity of A-L has not been assessed on various *Plasmodium falciparum Kelch 13* and *Pfmdr1* genes. Therefore, this study sought to determine the therapeutic efficacy of A-L on *P. falciparum* parasites isolated from Ghana.

**Methods:**

The clinical study was done in Ga West Municipality, Ghana, where 78 uncomplicated malaria patients were recruited with prior consent. The patients were treated orally with A-L according to national treatment guidelines. Baseline parasitaemia was determined before treatment and 8-hourly parasitaemia posttreatment were determined till initial clearance of parasitaemia and at days 7, 14, 21, and 28. *Kelch 13* and *Pfmdr1* genes were genotyped by sequencing using baseline samples. Parasite clearance characteristics were determined using Parasite Clearance Estimator beta 0.9 application.

**Results:**

Five *Kelch 13* (F446I, S466N, R539I, A578S, and A676S) and three *Pfmdr1* mutations (N86Y, Y184F and D1246Y) were identified in 78 infected samples. About 8% of the samples contained two *Pfmdr1* double mutations (N86Y & D1246Y and Y184F & N86Y). Additionally, three samples (3.8%) were found to contain both *Kelch 13* mutations and *Pfmdr1* wild type genes. In all patients, parasitaemia persisted within the first 24 h of A-L therapy. However, at hour 40, only two patients were parasitaemic while all patients were aparasitaemic at hour 48. The genotypic profiles of the two persistent parasites at hour 40 were F446I and D1246Y, and R539I, Y184F, and N86Y. The slope half-life of the former was 6.4 h while the latter was 6.9 h and their respective PCT99 were 47.9 h and 49.2 h as well as a clearance rate constants of 0.109 and 0.092 respectively.

**Conclusion:**

This study reports the effectiveness of A-L on various *P. falciparum* mutant alleles. However, continuous surveillance of *Kelch 13* mutations and *Pfmdr1* gene in Ghana and regular assessment of the therapeutic efficacy of A-L and other artemisinin derivatives is recommended.

## 1 Introduction

Malaria is a major public health concern and a leading cause of death globally. The World Health Organization (WHO) estimated that 228 million cases of malaria occurred worldwide and approximately 405,000 deaths were due to malaria in 2018 (WHO, 2019). In sub-Saharan Africa (SSA) of which Ghana is a part, malaria is still a serious health challenge. The proportion of out-patient department (OPD) cases attributable to malaria in Ghana decreased from 43.7% to 30.9% in 2014 and increased again to 38.7% in 2016 and further dropped to 34.0% in 2017 (Ghana Health Service, 2018). Although there are fluctuations in these figures, Ghana is still considered heavily burdened by malaria.

Artemisinin-based Combination Therapy (ACT) has been the treatment option for uncomplicated malaria in Ghana since 2005 (Koram et al., 2008). This change was necessitated by widespread treatment failures associated with chloroquine and sulfadoxine-pyrimethamine. Artesunate-Amodiaquine (AS-AQ) was then selected as the first-line drug. However, AS-AQ became unpopular due to adverse drug reactions and safety concerns that were reported across the country (Akpalu Jr et al., 2005) and elsewhere (Hatton et al., 1986; Neftel et al., 1986). Subsequently, Ghana reviewed its anti-malaria drug policy and introduced two additional ACTs, namely; Artemether-Lumefantrine (A-L) and Dihydroartemisinin-Piperaquine (DHAP) (Ministry of Health, 2009)). Afterwards, A-L became the first-line drug of choice due to its proven efficacy and host tolerability (Bharti et al., 2016).

A-L is a combination of fast-acting, artemether and long-acting lumefantrine (an arylamino alcohol related to quinine, mefloquine and halofantrine) in commercially available fixed dose combinations. Artemether quickly reduces *Plasmodium* parasitaemia with resolution of clinical symptoms, while long-acting lumefantrine prevents recrudescence of the parasite (Premji, 2009). A-L ultimately reduces the pressure on the parasite to develop resistance and it has been the first-line treatment in several malaria endemic countries (Teklemariam et al., 2017; Pfeil et al., 2015; Kabanywanyi et al., 2010).

Artemether-Lumefantrine is very effective in clearing parasites especially in Africa. Whereas there has been a drastic reduction in the proportion of deaths attributable to malaria from 4.2% in 2016 to 2.0% in 2017, the prevalence of malaria in Ghana is still very high (2017 prevalence was 20.3%) (Ghana Health Service, 2018). Even although derivatives of artemisinin are still efficacious in treating both uncomplicated (Plucinski et al., 2017) and severe malaria (Dondorp et al., 2010), treatment failure has only been observed when there are both artemisinin and partner drug resistance. Studies done in Greater Mekong Subregion, namely, Thailand, Cambodia, Laos, Myanmar, Vietnam, and China, have reported prolonged parasite clearance of *P. falciparum* following artemisinin therapy (Wongsrichanalai and S. R. Meshnick SR., 2008; Ariey et al., 2014; Phyo et al., n.d.; Thanh et al., 2017) as well as artemisinin resistance occurring along the Cambodia–Thailand border, the same area where chloroquine resistance spread to other parts of the world some decades ago (Dondorp et al., 2009; Noedl et al., 2008). Unfortunately, there is a simultaneous emergence of artemisinin partner drug resistance such as mefloquine and piperaquine, which has resulted in treatment failure rates along Cambodian-Thai, Cambodia-Laos, and Thai-Myanmar borders (Phyo et al., n.d.). In these studies, mutations in *Kelch13* propeller region were associated with artemisinin resistance (Ariey et al., 2014). *Kelch 13* propeller gene mutations recently reported to be associated with artemisinin resistance were F446I, N548Y, N548I, M476I, M476V, Y93H, R539T,P553L, R561H and C580Y (Chhibber-Goel and Sharma, 2019).

Due to the high rate of polymorphism in *Kelch 13* and the possible importation of *P. falciparum* with low sensitivity to ACTs from Southeast Asia, it is important to regularly assess the efficacy of first-line anti-malaria treatment regimen against *P. falciparum* parasites circulating in a defined endemic area for efficient management of malaria. Hence, the objective of the study was to assess the impact of *Kelch 13* mutations on the efficacy of artemether-lumefantrine in the Ga West Municipality in the Greater Accra Region of Ghana. Moreover the study evaluated the compliance of patients and/or guardians to the treatment plan and the efficacy of 6-dose A-L regimen in the treatment of uncomplicated malaria.

## 2 Materials and methods

### 2.1 Ethics approval and consent to participate

Ethics and protocol approval for this study was granted by Ghana Health Service Ethical Review Committee (GHS-REC002/03/18). Written consent to participate in this study was sort from guardians of minor patients (5–18 years) and patients >18 years consented for themselves.

### 2.2 Study design and sample collection sites

**Fig. 1:**
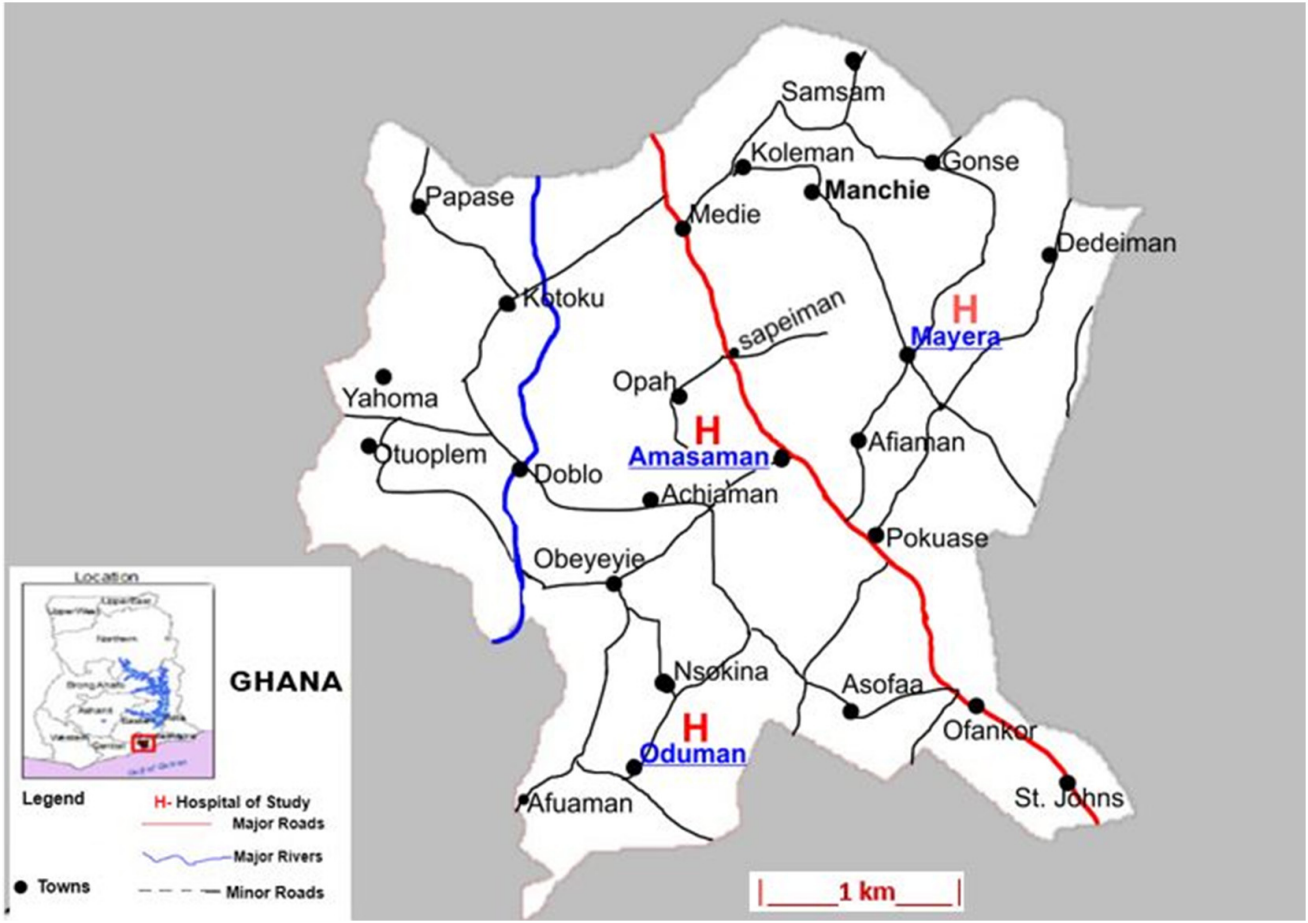
Map of Ga West Municipality indicating the study sites.

This clinical study was carried out in three health centres in Ga West Municipality (5°42′9″N, 0°18′0″W) in the Greater Region (5.8143°N, 0.0747°E) of Ghana; Ga West Municipal Hospital, Amasaman (5.7020708, −0.2992889), Oduman (5.64171, −0.3302) and Mayera (5.720578, −0.2703561) health centres. Sample collection was done between March and October 2018. Ga West Municipal Hospital, located in the Municipal capital, Amasaman, an urban community, is a 70-bed facility with 24-h operations, While Oduman and Mayera health centres are rural-sited health facilities (Fig. 1) with 22- and 15-bed facilities respectively.

### 2.3 Inclusion and exclusion criteria

Patients (aged ≥5 years, body weight ≥ 15 kg, blood type: O positive, sickle cell test: negative) with uncomplicated malaria (confirmed by microscopy) who consented to provide 8 hourly blood samples (about 50 μL) for 72 h or more were included in the study. All patients received Artemether-Lumefantrine therapy and none received any parenteral fluid during the study period. In addition, patients included in this study presented with one or more classical signs of malaria (fever, chills, headache, sweating, vomiting, etc.). There was no minimum or maximum parasitaemia set to be included in the study. Exclusion criteria were patients younger than 5 years, those with a history of liver abnormalities, and prior treatment with any anti-malarial. Additionally patients whose residences were more than 5 miles (8 km) farther from the health facilities they sorted treatment were excluded.

### 2.4 Sample size determination

The minimum number of *P. falciparum* infected patients to be recruited for a single-arm efficacy study was based on the revised WHO protocol (Methods for surveillance of antimalarial drug efficacy, 2009). Using the day 28 the cure rate of A-L to be 99.2% as determined recently in Ghana (Abuaku et al., 2019), a 95% confidence level, and 5% margin of error, the minimum calculated sample size was 73.

### 2.5 Home verification of consented patients and follow-up strategy

On the day the patients were discharged from the health facility, the research team members followed the patients to their homes. The digital address of the place was taken using the GhanaPost digital address application as well as Google map coordinates. Again, all landmarks that identified their residences were also recorded as well as the telephone numbers of patients over 18 years or that of their guardian, if the patient was under 18 years.

### 2.6 Follow-up sampling

Patients were followed up 8 hourly till initial clearance of parasitaemia and at days 7, 14, 21, and 28. Follow-up samples were collected using sterile 70% alcohol swabs, blood lancets, paediatric EDTA tubes (BD Microtainer, Frankyn Lakes, NJ, USA), miniature sterile plastic pipettes, cotton wool balls and phlebotomy plaster. Alternate middle and ring fingers on the left and right hands were used during the follow-up sampling. The fingers were sterilised with the alcohol swab and with the lancets, a sharp prick was made on the finger. First drop of blood was wiped off with the cotton ball. A firm press yielded a bolus of blood and with two quick aspirations, approx. 100 μL of blood was taken into the paediatric EDTA tubes. The blood sample was adequately mixed with the anticoagulant. The tubes were then labelled with patient ID, date, and time of sample collection. The wound was dressed and covered with phlebotomy plaster.

### 2.7 Source and strength of Artemether-Lumefantrine

Three different brands of Artemether-Lumefantrine (Lonart, Coartem, and Artrifan) were supplied to each of the health facilities by the Government of Ghana, through the Central Medical Stores, Ghana during the routine supply of commodities to health facilities as part of the bulk distribution system of Ghana Health Service. The strength per tablet of each brand of Artemether-Lumefantrine was 20 mg of Artemether and 120 mg Lumefantrine in a combined dose.

### 2.8 Administration of Artemether-Lumefantrine anti-malaria drug

Baseline *P. falciparum* parasitaemia was recorded before the patients were treated orally with Artemether-Lumefantrine. Treatment protocol was in accordance with Ministry of Health guidelines for malaria case management in Ghana. Per protocol and practice in Ghana, the recommended treatment was a 6-dose regimen over a 3-day period based on the number of tablets per dose according to predefined weight bands (15–24 kg = 2 tablets, 25–34 kg =3 tablets and > 34 kg = 4 tablets). First dose was supervised by the treatment nurse while patients and/or guardians administered the rest of the dosage at home. Patients were advised to take the recommended dosages over the 72 h period as prescribed by the attending clinician.

### 2.9 *P. falciparum* parasitaemia determination

Three separate thick blood films were prepared using 4–5 μL of whole blood, stained with 3% Giemsa stain. Parasite densities were determined by two experienced malaria microscopists. A third opinion was sought in cases where a pair of parasite count differed by more than 10%. Presence of parasitaemia was assessed before the next sampling was due, however, parasite densities were determined later.

### 2.10 Preparation of dry blood spot for amplicon sequencing

Baseline and last parasite detectable blood samples (≈35 μL) were spotted on filter paper (Whatman™ Grade 31 ET CHR, Buckinghamshire, UK). Blood spots were dried and shipped to Wellcome Sanger Institute, UK, for selective whole genome sequencing.

### 2.11 *P. falciparum* genomic DNA extraction

Using a 3 mm diameter single-hole paper punch, 6–8 small pieces of dry blood spot (DBS) were cut into a 2 mL micro-centrifuge tubes from which DNA was extracted. DNA extraction was done using QIAamp DNA Investigator Kit (Qiagen, California, United States) following the kit manufacturer's instructions. At least 5 ng of DNA was used as template for genotyping by sequencing.

### 2.12 Selective whole genome amplification (sWGA)

Detailed *P. falciparum* primers designed to identify *P. falciparum* genes have been published (Oyola et al., 2016). Selective whole genome amplification (sWGA) reaction was performed in a 50 μL reaction volume containing at least 5 ng of template DNA, 1× BSA, 1 mM dNTPs, 2.5 μM of each amplification primer, 1× Phi29 reaction buffer and 30 units of Phi29 polymerase enzyme (New England Biolabs). Isothermal conditions were used for sWGA. The conditions were 35 °C for 5 min, 34 °C for 10 min, 33 °C for 15 min, 32 °C for 20 min, 31 °C for 30 min, 30 °C for 16 h then heating at 65 °C for 15 min to inactivate the enzymes prior to cooling to 4 °C. Subsequent to sWGA, the products were cleaned using Ampure XP beads after which the bead/DNA mixture was placed on a magnetic rack to capture the DNA-bound beads. Beads were washed twice with 80% ethanol and the bound DNA eluted with elution buffer. DNA libraries were prepared with the cleaned DNA products using NEBNext DNA sample preparation kit (New England Biolabs). DNA libraries were sequenced using Illumina HiSeq 2500 DNA Sequencer.

#### 2.12.1 Analysis of sequence reads

Only *P. falciparum* sequence reads were analysed with no human DNA reads involved in the process. *P. falciparum* sequence reads were automatically demultiplexed and fastq data files were generated using the onboard PC. Subsequently, Bioedit v7.2 was used to trim low-quality bases from their ends. Each dataset was analysed independently by mapping sequence reads to the *P. falciparum* 3D7 reference genome using Burrows-Wheeler Aligner (BWA). SNP analysis was performed on sequenced data targeting SNPs present in the core genome as well as key malaria drug resistance genes (*Pfmdr1* involved in multiple drugs and *Kelch 13* involved in artemisinin resistance).

### 2.13 Parasite clearance rate determination

Parasite clearance curves were derived from the parasite counts and parasite clearance half-life. The clearance rate constant and parasite clearing time were estimated using the Parasite Clearance Estimator beta 0.9 application developed by WorldWide Antimalarial Resistance Network (WWARN) in 2015. This tool estimates the parasite clearance rate constant based on the linear part of the log_e_ parasite density–time profile. Default functions were used for analyses.

## 3 Results

### 3.1 Outcome of case selection and patient follow-up

**Fig. 2:**
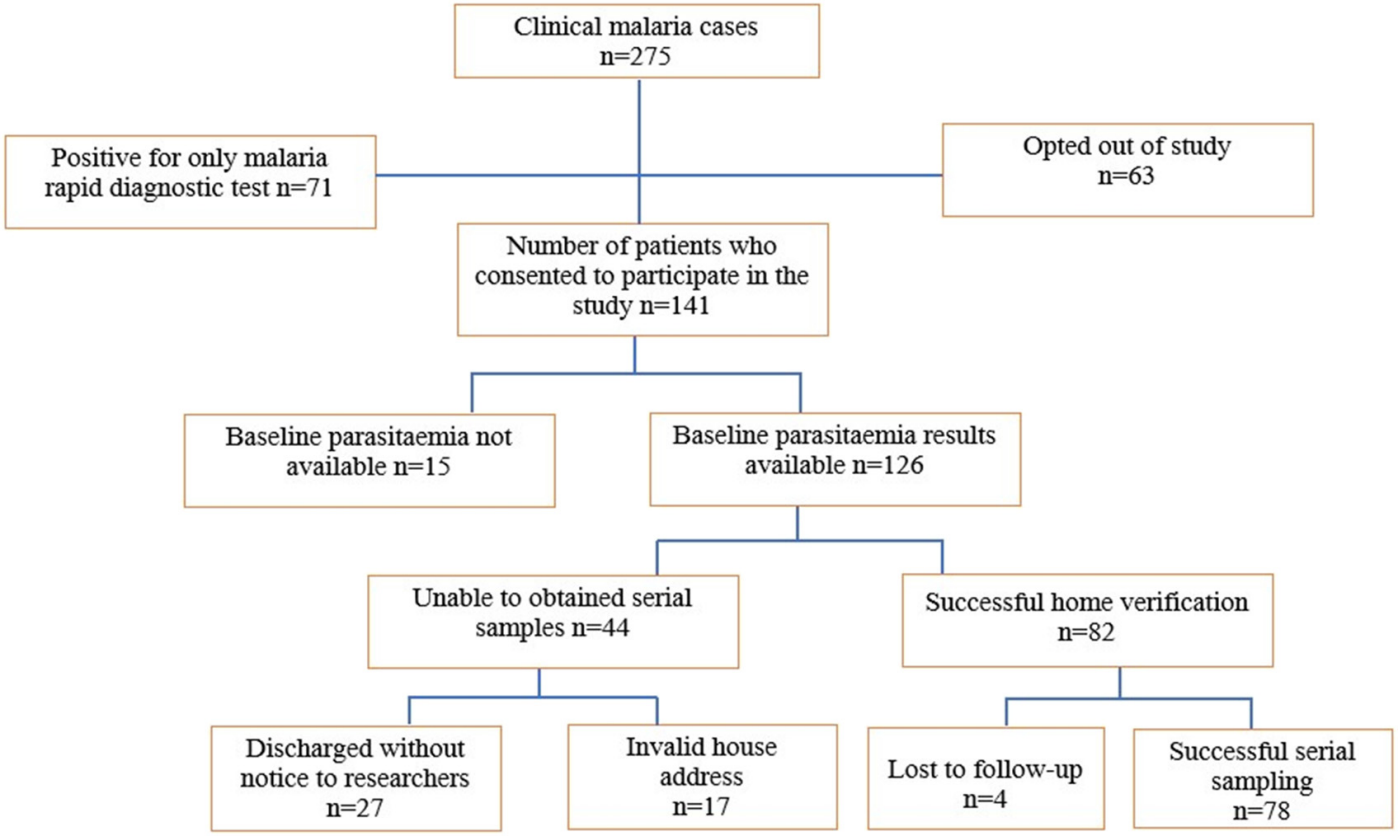
Flow chart for sample section and patient follow-up.

During the study period, 275 malaria patients were encountered. Of this number, microscopy did not detect 71 infections, however, rapid diagnostic testing did. Subsequently, 63 patients with microscopically detectable parasitaemia did not consent to take part in the study. Finally, 141 patients with *P. falciparum* parasitaemia consented to take part in the study. However, baseline parasitaemia for 15 patients was not available. These patients were excluded from the study. Of the 126 patients with baseline parasitaemia, 27 of them were treated and discharged without informing the research team and another group of 17 patients provided incomplete or invalid information, hence, their residence could not be traced for further sampling. Although 85 patients provided adequate information to aid home visits, seven of them were lost to follow-up or a particular timed sample could not be taken during treatment. Fig. 2 summarizes the patient sample sections and patient follow-up.

### 3.2 Characteristics of wild type and mutant strains identified

Table 1 represents the age, gender, and previous malaria treatment classifications of the 78 malaria patients (minimum and maximum parasitaemia were 12,798 and 165,405 parasites/μL respectively) who participated in the study. Most of the patients, 52 (66.7%) were 6–15 years old. Of these 52 patients, 31 were infected with the wild type while 21 were infected with various forms of the parasite mutant parasites. The rest, 26 patients were over 15 years (15 and 11 of these patients were infected with wild type and various forms of mutant parasites respectively). More females were infected 42 (53.8%). Of the 42 infected females, 26 were infected with wild type and 16 infected with various form of mutant parasites. In case, of the 36 male infectees, 20 were infected with wild type and 16 infected with various mutant parasites. Of the total participants, 19 (24.4%) were previously treated for laboratory confirmed malaria in the last 6 months, 16 (84.2%) of the 19 were found to be infected with various forms of mutant parasites while the other 3 (15.8%) *P. falciparum* infections were wild type. Twenty-four (30.7%) were also previously treated with laboratory confirmed malaria within the past 6 months to 1 year (8 were found to be infected with mutant parasites and 16 infected with wild type parasites). Finally, 35 (44.8%) of the total infectees were also previously treated for malaria within 1–2 years. Of this number, 8 were found to be infected with mutant parasites and 27 infected with wild type parasites. It was further observed that none of the malaria patients whose samples were analysed in this study had ever travelled to countries where parasites with reduced sensitivity to ACT were highly prevalent.

**Table 1:** Age, gender and history of previous malaria treatment of the participants.

	Wild type strains (*n* = 46)			Mutant strains (*n* = 32)			
Characteristics	GWMH (*n* = 22)	MHC (*n* = 17)	OHC (*n* = 7)	GWMH (*n* = 13)	MHC (n = 7)	OHC (*n* = 12)	Total
Age group							
6–15 years	16 (72.7%)	10 (58.8%)	5 (71.4%)	9 (69.2%)	4 (57.1%)	8 (66.7%)	52[31^a^+21^b^]
>15 years	6 (27.3%)	7 (41.2%)	2 (28.6%)	4 (30.8%)	3 (42.9%)	4 (33.35)	26[15^a^+11^b^]
Gender							
Male	9 (41.0%)	5 (29.4%)	6 (85.7%)	5 (38.5%)	2 (28.6%)	9 (75.0%)	36[20^a^+16^b^]
Female	13 (59.0%)	12 (70.6%)	1 (14.3%)	8 (61.5%)	5 (71.4%)	3 (25.0%)	42[26^a^+16^b^]
Last treated malaria							
0–0.5 years	0 (0.0%)	2 (11.7%)	1 (14.3%)	7 (53.8%)	4 (57.1%)	5 (41.7%)	19[3^a^+16^b^]
0.5-1 year	7 (31.8%)	7 (41.2%)	2 (28.6%)	4 (30.8%)	1 (14.2%)	3 (25.0%)	24[16^a^+8^b^]
1-2 years	15 (68.2%)	8 (47.1%)	4 (57.1%)	2 (15.4%)	2 (28.6%)	4 (33.3%)	35[27^a^+8^b^]
History of travel to regions with drug resistant *P. falciparum* strains							
Yes	0 (0.0%)	0 (0.0%)	0 (0.0%)	0 (0.0%)	0 (0.0%)	0 (0.0%)	0 (0.0%)
No	22 (100%)	17 (100%)	7 (100%)	13 (100%)	7 (100%)	12 (100%)	78 (100%)

a Sum of frequencies of wild type strains; ^b^ sum of frequencies of gene alleles. Ga West Municipal Hospital, Mayera Health Centre, Oduman Health Centre; SEA-South East Asia.

### 3.3 Persistence of *P. falciparum* parasitaemia following Artemether-Lumefantrine therapy

**Fig. 3:**
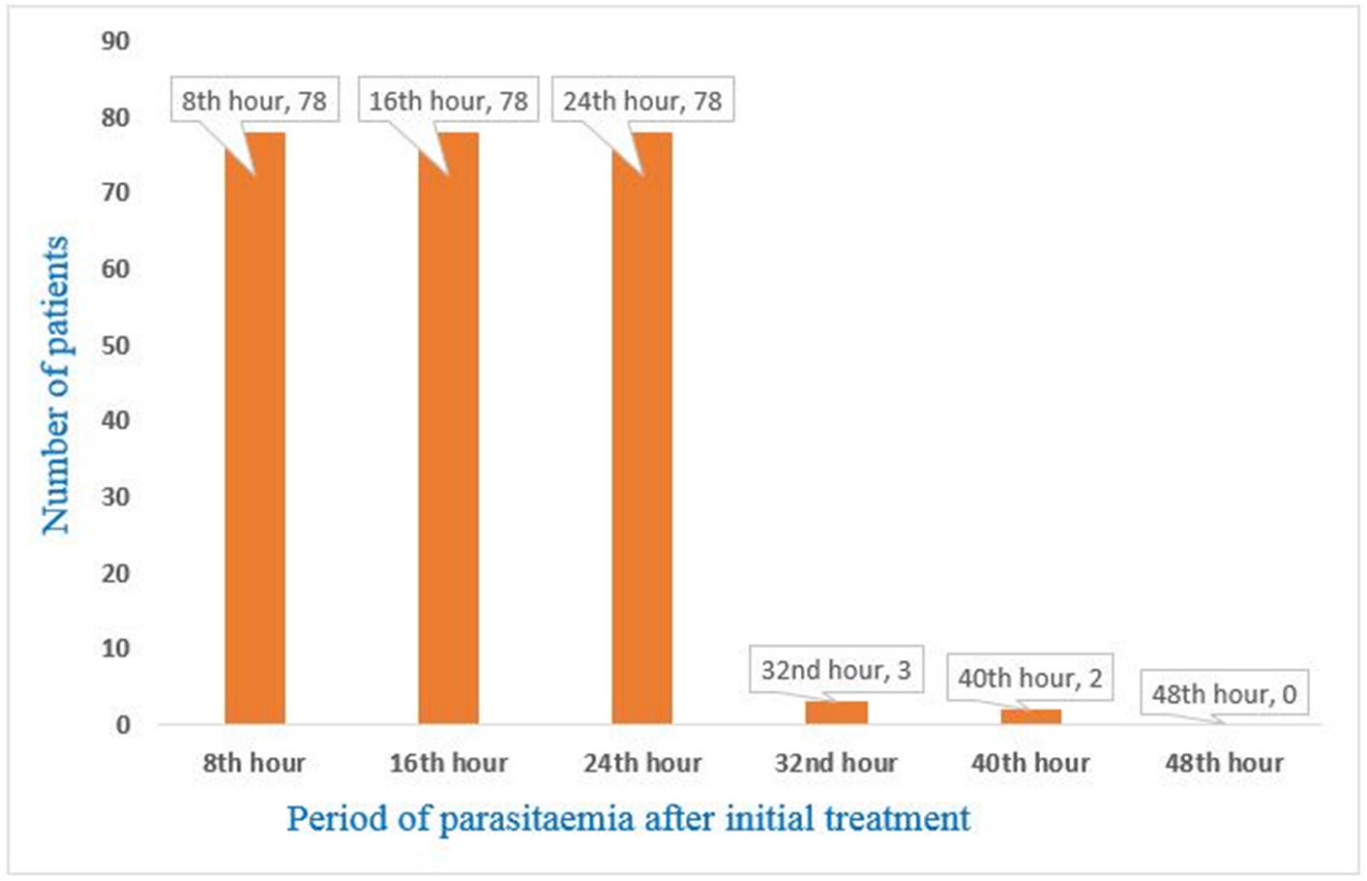
8-hourly parasitaemia following Artemether-Lumefantrine therapy.

*P. falciparum* parasitaemia was detected in all 78 patients from 8th hour to 24th hour after initial dosing. However, only three (3.8%) and two (2.6%) patients were parasitaemic at 32nd and 40th hour, respectively (Fig. 3). The two parasitaemic patients were females between 6 and 15 years and have had previous malaria within the last year. All patients were aparasitaemic after 40th hour of initial treatment.

### 3.4 Outcomes of patients' follow-up

Of the 78 patients, 41–69 patients were available for resampling on day 7 – day 28. All patients examined on and after day 7 were aparasitaemic. Patients unavailability, decline to provide samples and lost to follow-up were the main reasons all patients were not followed up from day 7 to day 28 (Table 2).

**Table 2:** Patients and parasitological outcome during follow-up.

Follow up time	8–40 h	48–56 h	Day 7	Day 14	Day 21	Day 28
Number of patients sampled	78 (100%)	76 (97.4%)	69 (88.5%)	59 (75.6%)	48 (61.5%)	41 (52.6%)
Malaria parasitaemia	100%	0%	0%	0%	0%	0%
Number of patients unable to re-sample	0 (0%)	2 (2.6%)	9 (11.5%)	19 (24.4%)	30 (38.5%)	37 (47.4%)
Reasons for inability to re-sample						
Unavailable at re-sampling time	0 (0%)	2 (100%)	5 (55.6%)	9 (47.4%)	15 (50.0%)	19 (51.3%)
Decline to provide sample	0 (0%)	0 (0%)	3 (33.3%)	5 (26.3%)	7 (23.3%)	8 (21.6%)
Lost to follow-up	0 (0%)	0 (0%)	1 (11.1%)	5 (26.3%)	8 (26.7%)	10 (27.0%)

### 3.5 Prevalence of *Kelch 13* and *Pfmdr1* mutations

The prevalence of mutant alleles identified in the *P. falciparum* genome is presented in Table 3. The overall prevalence of both *Kelch 13* mutations and *Pfmdr1* mutant alleles in the *P. falciparum* gene was 41.0%. More than half (63.2%) of the parasites isolated from Oduman Health Centre contained *Kelch 13* mutations and *Pfmdr1* mutant alleles, while parasites isolated from Ga West Municipal Hospital and Mayera Health Centre contained 37.1% and 29.2% respectively. Five different nonsynonymous *Kelch 13* mutations were identified; F446I, S466N, A578S, R539I, and A676S. The most prevalent *Kelch 13* mutation was R539I (6.4%). R539I mutant was identified separately in 3 samples and in combination with Y184F and D1246Y *Pfmdr1* mutants in 2 samples. It is worth noting that R539I mutant was identified in specimens collected from all three study sites. However, its combination with *Pfmdr1* mutant allele was not identified in parasites isolated from Mayera Health Centre. The most prevalent *Pfmdr1* gene mutation was D1246Y (7.7%), and like R539I mutant, it was identified in all study sites, however it was more prevalent in Ga West Municipal Hospital than the other two sites. Two double *Pfmdr1* mutants, N86Y & D1246Y and Y184F & N86Y were identified with overall respective prevalence of 3.8%. N86Y & D1246Y double mutants were identified in all study sites, but Y184F & N86Y mutants were identified in three parasites isolated in Oduman Health Centre. Interestingly, both *Kelch 13* and *Pfmdr1* gene polymorphisms were identified in five (6.4%) samples. Except for F446I and D1246Y mutants that were identified in two study sites, the other mixed mutants were all identified from only one site. Additionally, both *Kelch 13* mutations and *Pfmdr1* wild type were observed in three samples; one sample contained S466N and wild type *Pfmdr1* while two samples were found to contain A676S and WT *Pfmdr1* (N86, Y184, D1246)*.*

**Table 3:** Frequencies of non-synonymous mutations in Ga West Municipality, Ghana.

	Parasite strain	Overall	GWMH	MHC	OHC
Gene	Wild type strain	46 (59.0%)	22 (62.9%)	17 (70.8%)	7 (36.8%)
*Kelch 13* gene	A578S	1 (1.3%)	0 (0.0%)	0 (0.0%)	1 (5.3%)
	R539I	3 (3.8%)	1 (2.9%)	1 (4.2%)	1 (5.3%)
	A676S	2 (2.6%)	1 (2.9%)	0 (0.0%)	1 (5.3%)
*Pfmdr1* gene	N86Y	4 (5.1%)	3 (8.6%)	0 (0.0%)	1 (5.3%)
	Y184F	4 (5.1%)	1 (2.9%)	1 (4.2%)	2 (10.5%)
	D1246Y	6 (7.7%)	3 (8.6%)	2 (8.3%)	1 (5.3%)
	N86Y & D1246Y	3 (3.8%)	1 (2.9%)	1 (4.2%)	1 (5.3%)
	Y184F & N86Y	3 (3.8%)	0 (0.0%)	0 (0.0%)	3 (15.7%)
*Kelch 13* and *Pfmdr1* gene polymorphisms					
	F446I and D1246Y	2 (2.6%)	1 (2.9%)	1 (4.2%)	0 (0.0%)
	A578S and N86Y	1 (1.3%)	1 (2.9%)	0 (0.0%)	0 (0.0%)
	R539I and D1246Y	1 (1.3%)	1 (2.9%)	0 (0.0%)	0 (0.0%)
	R539I, Y184F & N86Y	1 (1.3%)	0 (0.0%)	0 (0.0%)	1 (5.3%)
*Kelch 13* mutation and *Pfmdr1* wild type					
	S466N and WT *Pfmdr1*	1 (1.3%)	0 (0.0%)	1 (4.2%)	0 (0.0%)
	A676S and WT *Pfmdr1*	2 (2.6%)	1 (2.9%)	0 (0.0%)	1 (5.3%)
	Total	78	35	24	19

GWMH-Ga West Municipal Hospital, MHC-Mayera Health Centre, OHC-Oduman Health Centre, WT-Wild type.

### 3.6 Pharmaco-activity of Artemether-Lumefantrine (A-L) on different *P. falciparum* genes

Table 4 compares the pharmaco-activity of standard Artemether-Lumefantrine (A-L) treatment based on parasite clearance rate constant (CRC), slope half-life and parasite clearance times (PCTs) of wild type and mutant *P. falciparum* parasites. The mean wild type parasite clearance rate constant (CRC) (defined as the estimated fraction by which parasitaemia is reduced per hour) was higher (0.231/h) than the CRC of *Kelch 13* and *Pfmdr1* mutants, and parasites having both *Kelch 13* and *Pfmdr1* gene mutants. Despite been lower, the mean CRC of R539I, Y184F, N86Y, and A578S and N86Y were over 0.2/h. These values were marginally lower than that of the wild type. The other parasites had CRC less than 0.2/h with that of R539I, Y184F & N86Y being the lowest (0.092/h). The mean slope half-life (defined as the estimated time taken for parasitaemia to reduce by half) of the wild type was 4.3 h. This value was equal to the mean half-life of N86Y and slightly lower than the mean half-life of R539I (4.8 h), Y184F (4.5 h), D1246Y (4.8 h), N86Y & D1246Y (4.7 h), Y184F & N86Y (4.5 h) and the half-life of A578S (4.5 h) and A578S & N86Y (4.4 h). However, it is noteworthy that the mean half-lives of F446I & D1246Y and the half-lives of A578S & N86Y, R539I & D1246Y and R539I, Y184F & N86Y were greater than 5 h (the WHO benchmark for determination of delayed parasite clearance). Parasite clearance time (PCT50), defined as the estimated time in hours for parasitaemia to reduce by 50% of the initial value, as well as PCT90, PCT95 and PCT99 were also estimated. The mean PCT50 of the wild type was 11.5 h, a value only higher than PCT50 of R539I & D1246Y (9.2 h) and D1246Y (10.9 h). All other *P. falciparum* parasites had PCT50 higher than that of the wild type. The mean PCT99 of the wild type was 28.8 h. Comparing to the other parasites, there was no significant difference in PCT99 among the parasites (variation of 1.7–4.4 h) except R539I, Y184F & N86Y and F446I & D1246Y mutants that recorded an estimated PCT99 of 49.2 h and 47.9 h respectively. The slope half-lives of *Kelch 13* S466N/WT *Pfmdr1* and *Kelch 13* A676S/WT *Pfmdr1* were 5.2 and 4.5 h respectively. Whereas the PCT50 of S466N/WT *Pfmdr1* was lower than A676S/WT *Pfmdr1,* PCT995, and PCT99 of S466N/WT *Pfmdr1* were higher than that of A676S/WT *Pfmdr1.*

**Table 4:** Comparative efficacy of A-L on wild type and *P. falciparum* gene alleles.

*P. falciparum* strains	Sample(s) analysed with PCE*	^1^CRC	^2^Slope half-life (hrs)	^3^PCT50 (hrs)	^4^PCT90 (hrs)	^5^PCT95 (hrs)	^6^PCT99 (hrs)
Wild type strain	46	0.231	4.3	11.5	19.1	22.1	28.8
*Kelch 13* gene mutations							
R539I	3	0.214	4.8	12.1	20.1	23.5	31.6
A578S	1	0.199	4.5	12.8	20.9	24.4	32.5
*Pfmdr1* gene mutations							
Y184F	4	0.201	4.5	14.1	22.1	25.5	33.1
N86Y	4	0.212	4.3	12.1	19.7	22.9	30.5
D1246Y	6	0.183	4.8	10.9	19.7	23.5	32.4
N86Y & D1246Y	3	0.186	4.7	11.4	20.2	23.7	32.4
Y184F & N86Y	3	0.197	4.5	11.8	19.9	23.5	31.6
*Kelch 13* and *Pfmdr1* gene polymorphisms							
F446I & D1246Y	2	0.109	6.4	13.7	27.1	35.5	47.9
A578S & N86Y	1	0.203	4.4	11.3	19.2	22.7	30.6
R539I & D1246Y	1	0.160	5.3	9.2	19.2	23.6	32.6
R539I, Y184F & N86Y	1	0.092	6.9	15.1	29.4	38.6	49.2
*Kelch 13* mutation and *Pfmdr1* wild type							
S466N and WT *Pfmdr1*	1	0.175	5.2	11.8	21.3	25.6	33.2
A676S and WT *Pfmdr1*	2	0.204	4.5	13.1	22.7	24.9	30.9

* PCE-parasite clearance estimator beta 0.9 application, ^1^ CRC-Clearance rate constant is fraction of parasites cleared from peripheral blood in 1 h; ^2^Slope half-life was the time taken for parasitaemia to reduce by half; ^3-6^PCTx is the time taken for parasitaemia to reduce by x% of the baseline parasitaemia. All figures are presented as mean values except in samples with single occurrence.

## 4 Discussion

In this study, five different *Kelch 13* mutations and three different *Pfmdr1* gene mutant alleles were identified in *P. falciparum* isolated from Ghana. Additionally, some *Pfmdr1* double mutations as well as both *Pfmdr1*/*Kelch 13* gene mutations and *Kelch 13* mutation/*Pfmdr1* wild type were identified. In all identified genes, the slope half-lives were less than 5 h except in *Kelch 13* S466N/ wild type (WT), *Pfmdr1* (N86, Y184, D1246), both *Kelch 13* and *Pfmdr1* mutants (F446I & D1246Y, R539I & D1246Y) and one *Kelch 13* and double *Pfmdr1* mutants (R539I, Y184F & N86Y) where estimated slope half-live were between 5.2 and 6.9 h. Meanwhile, one of the indicators recommended by Global Malaria Programme of World Health Organization to be used to determine delayed parasite clearance or artemisinin resistance is slope half-life. Parasites with slope half-life greater than 5 h are said to be responding slowly to ACT (Global Malaria Programme, 2016).

Another indicator recommended by WHO to be used to determine delayed parasite clearance and/or drug resistant *P. falciparum* was positive parasitaemia on day 3 (72 h) post ACT therapy (WHO, 2016). In all the infected patients evaluated, parasitaemia was not detected beyond hour 72, however, only two (2.6%) patients were parasitaemic at 40 h after A-L therapy. The PCT99 of these two persistent parasites (F446I & D1246Y, and R539I, Y184F & N86Y) were 47.9 h and 49.2 h respectively. Once these parasites were not detected at hour 72, we confirm their susceptibility to A-L.

The wild type *Pfmdr1* genes (N86 and D1246) have been shown to be associated with increased risk of treatment failure and decreased sensitivity to lumefantrine (Venkatesan et al., 2014; Mwai et al., 2009; Tumwebaze et al., 2015; Achol et al., 2019) but susceptible to aminoquinolines (Tumwebaze et al., 2015). In contrast, *Pfmdr1* D1246Y mutations has been reported to be susceptible to mefloquine, halofantrine, and artemisinin derivatives (Humphreys et al., 2007; Lekostaj et al., 2008; Sidhu et al., 2005). This study could not assess the efficacy of A-L on *Pfmdr1* wild type gene in isolation since it co-existed with either wild type *Kelch 13* or *Kelch 13* mutations. However, this study found the slope half-life of *Kelch 13* mutant S466N/WT *Pfmdr1* to be 5.2 h while *Kelch 13* mutant A676S/WT *Pfmdr1* was 4.5 h. Even although the slope half-life of the former was marginally above WHO benchmark of 5 h, the former was lower. At hour 33 after A-L treatment, 99% of baseline parasitaemia of S466N/WT *Pfmdr1* parasites was cleared. Even though the wild type *Pfmdr1* gene has been reported to respond slowly to A-L, the reverse was observed in this study. Furthermore, the previous finding that *Pfmdr1* D1246Y mutation is susceptible to artemisinin derivatives was corroborated by this study. Even though the slope half-life obtained for *Pfmdr1* D1246Y, *Pfmdr1* N86Y/D1246Y, *Kelch 13* F446I/*Pfmdr1* D1246Y and *Kelch 13* R539I/*Pfmdr1* D1246Y mutations were 4.8 h, 4.7 h, 5.3 h and 6.4 h respectively, their respective PCT99 were below 48 h. In Ghana, N86Y, Y184F, and D1246Y mutants have been previously reported (Asare et al., 2017; Kwansa-Bentum et al., 2011). These mutant parasites, which are susceptible to ACT, are very prevalent in the Central and its contiguous region, the Greater Accra. Parasites with one or more mutations in *Pfmdr1* gene were found to be susceptible to A-L, an observation that has already been reported (Venkatesan et al., 2014; Mwai et al., 2009; Achol et al., 2019; Sidhu et al., 2005). According to the findings from this study, parasitaemia for parasites with 184F, 86Y, 1246Y, 86Y & 1246Y, and 184F & 86Y genes halved every 4.3–4.8 h and by 30.5–33.1 h after A-L therapy, 99% of baseline parasitaemia was cleared.

Only one sample with three mutations (R539I, 184F & 86Y) was identified in this study. These parasites were identified in a patient resident in Nsakina, a rural community about 1.1 km east of Oduman Health Centre. Information on previous identification of R539I 184F & 86Y mutants in a single sample in Ghana and elsewhere was not readily available at the time of this publication. However, this parasite may not be uncommon since high prevalence of individual mutations, R539I, 184F, and 86Y, has been reported in several studies. The susceptibility of R539I, Y184F & N86Y triple mutations to A-L was similar to that of F446I & 1246Y. The co-presence of *Pfmdr1* 86Y and 1246Y genes, which are susceptible to lumefantrine and artemisinin derivatives, respectively, may have contributed to the overall susceptibility of the parasites to A-L. Therefore, it was surprising that the slope half-life of R539I, 184F & 86Y was 6.9 h, but the PCT99 value of 49.2 h obtained for this parasite was desirable. It must be stated that several factors affect the efficacy of oral medications. Host factors such as age, sex, physiological state, diseases of other organ systems, nutritional status, genetic disorders, and tolerance have been identified and reported to affect the efficacy of oral medication (Milanka et al., 2015). Even though the influences of these factors were not investigated, they could contribute to the observed prolongation of slope halves.

*Kelch 13* R539I identified in this study has been previously detected in several areas in Ghana. Previously, *Kelch 13* R539I has been reported in Hohoe (Volta region), Begoro (Eastern region), Sunyani (Bono region) and Wa (Upper West region). Variants of R539I namely, I543S and I543V have also been isolated from Navrongo (Upper East region), Sunyani and Hohoe (Matrevi et al., 2019). In this study, R539I mutation was found to respond to A-L very well. Slope half-life and PCT99 obtained for this mutation was 4.8 h and 31.6 h, respectively. Additionally, *Kelch 13* mutant S466N was initially reported in Columbia (Aponte et al., 2017) and subsequently in forest and coastal savannah zones in Ghana (Matrevi et al., 2019). However, this mutant gene has not been associated with artemisinin resistance, neither has it been found to reduce the sensitivity of ACT. The current study found the response of *Kelch 13* A676S mutation to A-L to be desirable. The parasite clearance times obtained for this mutation indicated high susceptibility to A-L.

In MalariaGEN *P. falciparum* Community Project surveillance on *Kelch, 13* SNPs involving 3411 clinical samples of *P. falciparum* obtained from 43 locations in 23 countries, of the several *Kelch 13* mutations identified, the key among them was A578S (Amato et al., 2016). In that study, A578S was reported to have not been validated as an artemisinin resistance marker. As was published by MalariaGEN, this study found A-L to be very effective against A578S. *Kelch 13* A578S mutant has been previously identified in studies that analysed *P. falciparum* samples from 2013 to 2019 in Ghana (Matrevi et al., 2019; Feng et al., 2019). Similar to this study, low frequency of F446I was seen in India, Myanmar, China, and Thailand (Nyunt et al., 2015; Wang et al., 2015; Ye et al., 2016). In a therapeutic study in China, the F446I mutation was found to be associated with delayed parasite clearance (Torrentino et al., 2014) and an intermediate rate of parasite clearance was found in clinical samples in Myanmar (Tun et al., 2016) but not in India (Feng et al., 2019). In this study, the effect of ACT on *Kelch 13* F446I could not be assessed in isolation because all the two F446I identified in this study was seen together with *Pfdmr1* 1246Y. Even though *Pfmdr1* 1246Y mutation is sensitive to lumefantrine, slope half-life of 6.4 h as well as PCT99 of 47.9 h was obtained, a result similar to what was reported in Myanmar. Notwithstanding, this parasite was not detected beyond 48 h, even though the slope half-life was 1.4 h above the WHO benchmark value. One of the two parasites was identified in a patient from Ga West Municipal Hospital, Amasaman and the other from Mayera Health Centre in Mayera. Interestingly, both patients resided in Afiaman in the Ga West Municipality in Ghana. The distance between their residences was approximately 0.5 km. Based on this observation, there could be a possibility that these parasites were circulating in Afiaman and its environs. Upscale surveillance for these mutations and *Pfmdr1* alleles in the community is therefore recommended since it has the potential of reducing the sensitivity of A-L.

## 5 Conclusion

Findings obtained from this study underscored the effectiveness of A-L on different parasite genes collected from the Ga West Municipality, Ghana. Except for parasites with these genes; F446I & 1246Y; R539I & 1246Y; R539I, 184F & 86Y; and S466N and WT *Pfmdr1* (N86, Y184, D1246) that recorded slope half-lives between 5.2 and 6.9 h, the slope half-lives of the rest of the parasites were less than the WHO benchmark of 5 h. In spite of the differences in their slope half-lives, all study patients were aparasitaemic by hour 40 after A-L treatment. Continuous assessment of the therapeutic efficacy of A-L and other artemisinin derivatives is, therefore, recommended.

## Authors' contributions

This study was conceptualised, designed, and coordinated by EA and DOA. Patient identification, sample collection, and determination of parasite counts were done by EA, CDT, and MO. EA, AB, and DOA analysed and interpreted the sequence data. The manuscript was drafted by EA and was proof-read by DOA, AB, CDT, and MO.

## Declaration of Competing Interest

The authors declare no conflict of interest.
